# Refolding Protocol of the Cysteine-Rich Domain of Frizzled8 from Inclusion Bodies

**DOI:** 10.3390/ijms27167198

**Published:** 2026-08-12

**Authors:** Ho-Jin Lee, Jie J. Zheng

**Affiliations:** 1 Department of Chemistry, School of Science and Technology, LeMoyne-Owen College, Memphis, TN 38126, USA; 2 Jules Stein Eye Institute, Department of Ophthalmology, David Geffen School of Medicine, University of California, Los Angeles, CA 90095, USA; 3 Molecular Biology Institute, University of California, Los Angeles, CA 90095, USA

**Keywords:** Frizzled8, cysteine-rich domain, inclusion bodies, protein refolding, disulfide bonds, HPLC purification, NMR spectroscopy, Wnt signaling, sFRP3

## Abstract

Properly folded proteins are essential for studying protein function and developing therapeutic applications. However, when recombinant proteins are overexpressed in bacterial systems such as *Escherichia coli*, they often accumulate as inclusion bodies, insoluble protein aggregates in non-native conformations. Here, we report a refolding protocol for mouse Frizzled8 cysteine-rich domain (mouse FZD8 CRD) recovered from inclusion bodies, yielding a soluble, folded CRD preparation suitable for NMR analysis. Because mouse FZD8 CRD contains multiple conserved cysteine residues that must form an appropriate intramolecular disulfide-bonding pattern, the refolding strategy combines denaturant-mediated solubilization, dilution into a redox-buffered refolding solution, concentration, dialysis, lyophilization, and HPLC purification. Lyophilization followed by HPLC purification enables the resolution of the desired CRD species from aggregated and misfolded products. In this workflow, HPLC served as a key purification step, providing the resolution needed to enrich a folded, multi-disulfide-bond-containing CRD species from closely related misfolded species. NMR spectroscopy supported the presence of a folded, conformationally homogeneous CRD preparation. This workflow was also applied to mouse secreted Frizzled-related protein 3 cysteine-rich domain (mouse sFRP3 CRD), suggesting that it may be useful for other Frizzled-type CRDs with conserved cysteine-rich architectures, although additional validation will be required before broader application to more distantly related cysteine-rich proteins.

## 1. Introduction

Frizzled receptors (FZDs) are a family of seven-transmembrane receptors that function as major cell-surface receptors for Wnt ligands [1,2,3]. In humans, the Frizzled family consists of 10 receptor subtypes, FZD1–FZD10, which share a conserved overall architecture but differ in ligand selectivity, tissue distribution, and signaling output. Aberrant expression or activation of FZD receptors has been reported in multiple human cancers and is associated with enhanced Wnt signaling [4,5,6,7,8,9].

**Figure 1:**
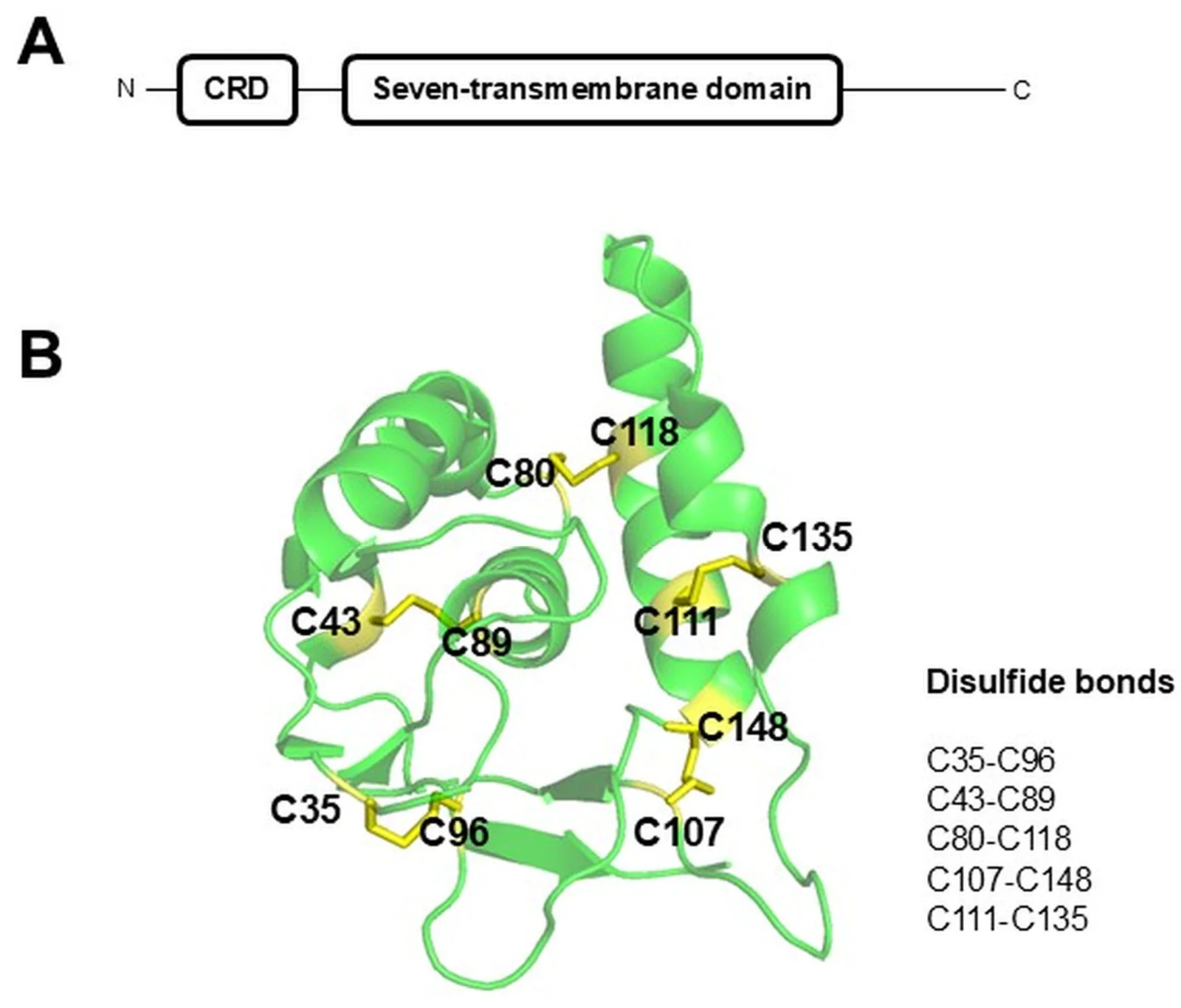
Structural features of Frizzled (FZD): (**A**) Schematic diagram of a Frizzled receptor, highlighting the extracellular cysteine-rich domain (CRD) and the seven-transmembrane domain. (**B**) Structure of mouse FZD8 CRD (PDB ID: 6TFM). The protein backbone is shown as a green cartoon representation, and disulfide bonds are shown as yellow stick models. Numbers shown in the structure indicate cysteine residue numbers that participate in intramolecular disulfide-bond formation. The molecular structure was generated using PyMOL 3.0.

FZD contains a cysteine-rich domain (CRD), a seven-transmembrane domain, and a cytoplasmic C-terminal region (Figure 1). Among these domains, the cysteine-rich domain (CRD) is the extracellular ligand-recognition module and directly participates in Wnt binding [10,11,12,13,14]. Previous structural studies of Wnt–Frizzled complexes have demonstrated that Wnt ligands engage the FZD CRD through defined protein–protein and lipid-mediated interactions [14,15]. Because the extracellular CRD mediates Wnt recognition at the receptor surface, the FZD CRD is expected to play an important role in regulating Wnt signaling and represents a useful target region for developing molecules that modulate Wnt–FZD interactions [16,17,18].

Structural studies reported that the FZD CRD contains 10 conserved cysteine residues, which form five intramolecular disulfide bonds in the native structure (Figure 1) [11,12,13,14,15]. This feature is critical for maintaining the correct three-dimensional fold required for Wnt ligand recognition.

To understand the structural features and develop small molecules or peptides targeting the CRD, we attempted to express mouse FZD8 CRD in *Escherichia coli* [17]. However, overexpressed mouse FZD8 CRD accumulates in inclusion bodies, which are typically insoluble, denatured, and trapped in non-native conformations. Common inclusion-body refolding strategies involve dissolving aggregated protein in high concentrations of denaturants and reducing agents to eliminate non-native disulfide bonds, followed by dilution or dialysis into conditions that support refolding and disulfide-bond formation [19,20,21,22,23]. In addition, on-column refolding has been proposed using size-exclusion, ion-exchange, and affinity chromatography [24,25,26,27].

For cysteine-rich proteins, the redox environment is particularly important because folding must permit disulfide reshuffling while favoring native connectivity. In principle, ten distinct cysteine residues can be paired into five disulfide bonds in 945 different intramolecular combinations, and only a small fraction corresponds to the native connectivity. This combinatorial complexity explains why cysteine-rich proteins, such as mouse FZD8 CRD, are susceptible to mispairing, aggregation, and non-native conformations during refolding. In this work, we describe a practical refolding protocol for mouse FZD8 CRD expressed in *E. coli* inclusion bodies. The workflow combines denaturant-mediated solubilization, reduction of non-native disulfide bonds, rapid dilution into an arginine-containing redox refolding buffer, concentration, dialysis, lyophilization, and HPLC purification. We also performed the refolding of mouse secreted Frizzled-related protein 3 cysteine-rich domain (mouse sFRP3 CRD), which contains 10 cysteine residues and shares structural similarity with Frizzled-type CRDs [28,29]. Like Frizzled CRDs, mouse sFRP3 CRD can bind Wnt ligands through a conserved Wnt-interacting surface. From the two independent studies, we suggest that this workflow may be useful for other cysteine-rich domains that are difficult to refold from inclusion bodies [30,31].

## 2. Results

### 2.1. Refolding Strategy for Mouse FZD8 CRD from Inclusion Bodies

Refolding recombinant proteins from inclusion bodies is a widely used strategy for recovering soluble proteins from bacterial expression systems [19,20,21]. The workflow described here builds on established dilution-based refolding approaches by adding downstream concentration, acidic dialysis, lyophilization, and RP-HPLC purification steps to reduce aggregation and enrich folded CRD species from a complex refolding mixture. The following are the refolding steps we used in this work.

Step 1. Solubilization and reduction of inclusion bodies: Inclusion bodies containing FZD8 CRD were obtained from *E. coli* cells grown in 4 L or 6 L cultures. After sonication, the inclusion-body fraction containing FZD8 CRD was collected as a pellet by centrifugation (SI Figure S1A). The pellet was washed with 20 mM Tris buffer, 1 M urea, pH 8.0, and centrifuged. The washed pellet was resuspended in 20 mL of denaturing buffer containing 8 M urea and 100 mM β-mercaptoethanol (SI Figure S1B). High-concentration urea was used to disrupt non-native hydrophobic interactions and unfold the aggregated protein, whereas β-mercaptoethanol was included to reduce pre-existing non-native disulfide bonds. The suspension was mixed thoroughly and incubated at room temperature for 1 h to maximize solubilization and reduction before clarification.

Step 2. Clarification of the denatured protein solution: The solubilized inclusion-body suspension was clarified by high-speed centrifugation (13,000 rpm for 1 h) to remove insoluble debris, genomic DNA, and other cellular contaminants. When necessary, centrifugation was repeated to obtain a clear denatured protein solution before initiating refolding.

Step 3. Oxidative refolding by rapid dilution: The clarified denatured FZD8 CRD solution was added dropwise directly into 3 L of refolding buffer containing 25 mM Tris, 50 mM arginine, and 10 mM oxidized glutathione (GSSG), pH 8.0. This dilution reduced the final urea concentration to approximately 0.05 M, creating conditions permissive for oxidative refolding while minimizing aggregation caused by locally high protein concentration. Arginine was included to suppress nonspecific aggregation, whereas GSSG provided an oxidizing environment to promote disulfide-bond formation [22,23]. After refolding, the 3 L solution was concentrated to approximately 100 mL at 4 °C using pressure-driven ultrafiltration in a stirred cell. The concentrated sample was dialyzed overnight at 4 °C against 5% acetic acid, during which aggregated and misfolded proteins precipitated. The precipitated material was removed by centrifugation, and the supernatant was frozen and lyophilized.

### 2.2. HPLC Purification and NMR Characterization of Folded Mouse FZD8 CRD

**Figure 2:**
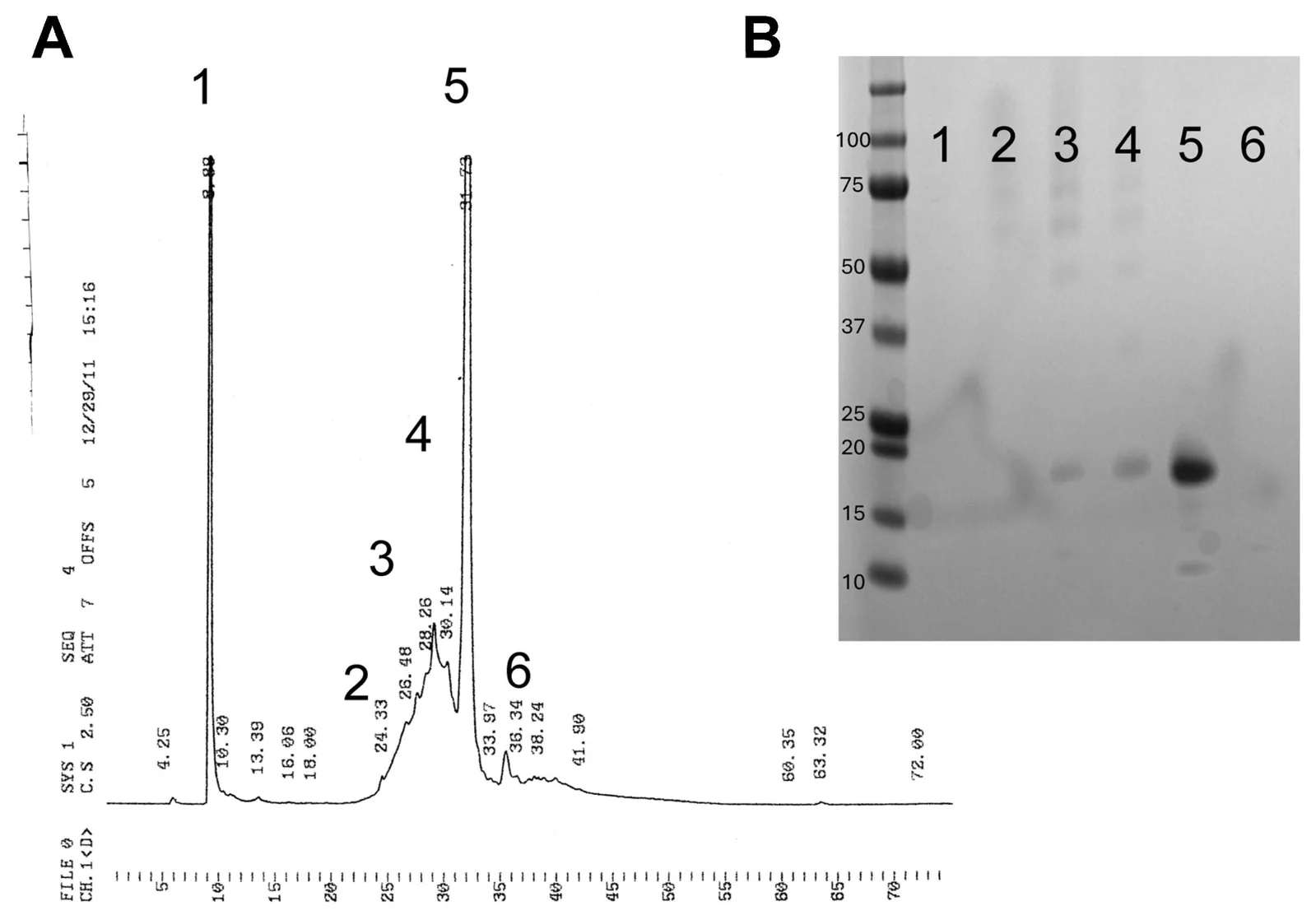
HPLC purification of refolded mouse FZD8 CRD (Ala^28^–Asp^155^) and analysis of collected fractions: (**A**) Representative HPLC elution profile of refolded mouse FZD8 CRD after dialysis and lyophilization. The numbered peaks or fractions in the chromatogram indicate samples collected during elution for further analysis. (**B**) SDS-PAGE analysis of the HPLC fractions corresponding to the numbers indicated in panel A. The first lane contains the protein marker, and the numbers indicate molecular weights in kDa. The gel was used to identify fractions enriched in the target protein, mouse FZD8 CRD, for subsequent pooling and characterization. Fraction 5, eluting at approximately 32 min, contained the target mouse FZD8 CRD and was used for subsequent dialysis and NMR analysis. Densitometric analysis of the SDS-PAGE gel in panel B estimated the purity of the HPLC-purified mouse FZD8 CRD fraction used for NMR analysis to be approximately 87%. Other peaks or fractions were not assigned molecular identities in this study and were excluded because SDS-PAGE did not show comparable enrichment of the expected mouse FZD8 CRD band.

**Figure 3:**
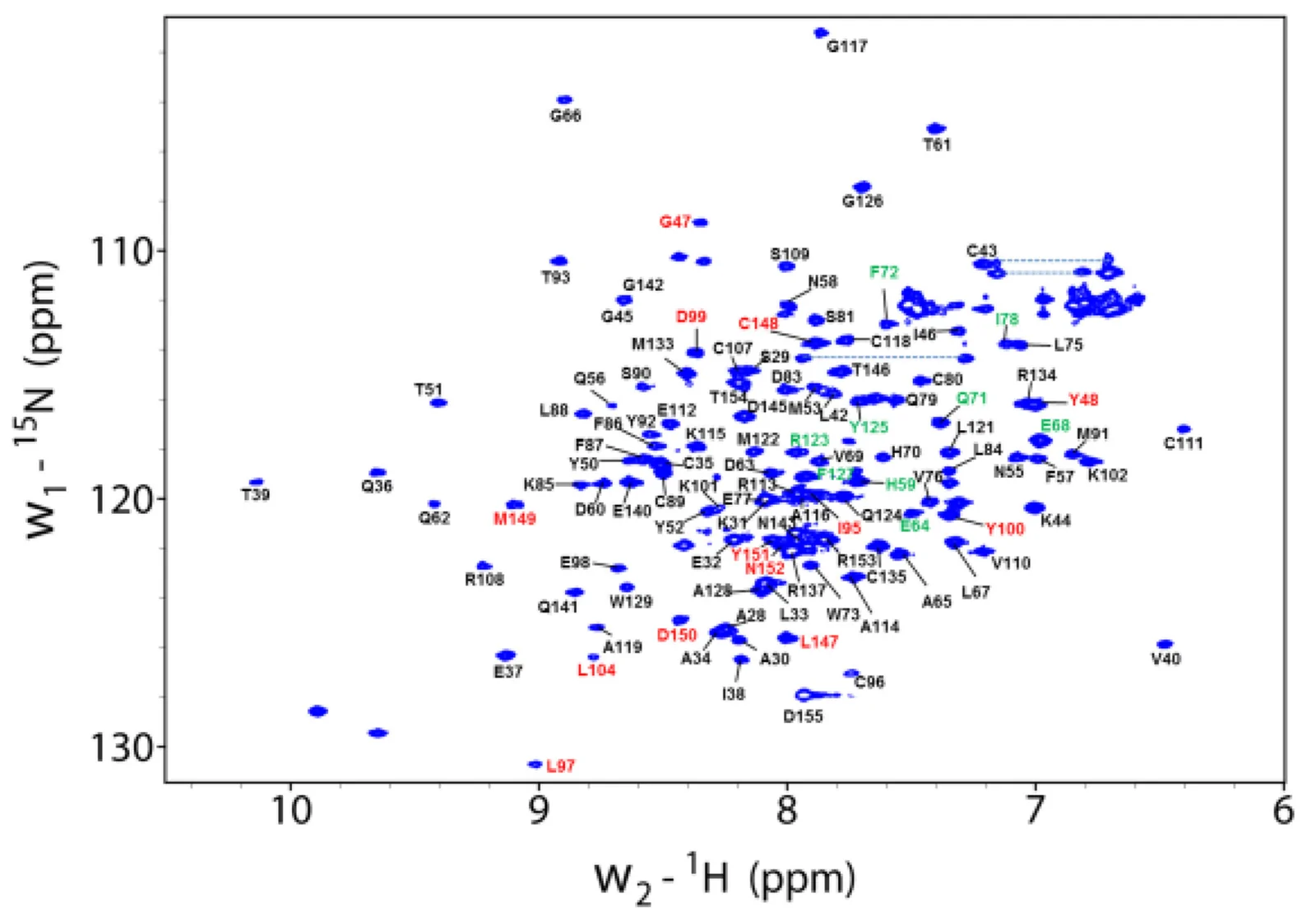
Two-dimensional ^1^H–^15^N HSQC spectrum of purified mouse FZD8 CRD (Ala^28^–Asp^155^). The spectrum was acquired using ~200 µM mouse FZD8 CRD in 50 mM phosphate buffer at pH 6.5 and shows well-dispersed amide resonances, consistent with a folded mouse FZD8 CRD structure. Mouse FZD8 CRD contains distinct lipid-binding and peptide-binding regions that contribute to Wnt recognition [14]. Assigned peaks corresponding to residues in the lipid-binding region are labeled in green, whereas assigned peaks corresponding to residues in the peptide-binding region are labeled in red. NMR data were processed using Bruker Topspin 3.0, and peak assignments were analyzed using CARA (computer-aided resonance assignment) [32] and Sparky [32].

The lyophilized sample containing FZD8 CRD was dissolved in 40 mL of water. Although a portion of the sample dissolved, some material remained insoluble, likely representing residual aggregates or insoluble impurities. The suspension was centrifuged at 4000 rpm for 20 min, and only the clarified supernatant was used for subsequent HPLC purification. A 5 mL aliquot of the clarified supernatant was injected onto a ZORBAX 300SB-C18 semi-preparative column (Agilent Technologies, Santa Clara, CA, USA) for reverse-phase HPLC purification at a flow rate of 2 mL/min using two solvents: solvent A, water containing 0.1% *v*/*v* trifluoroacetic acid, and solvent B, acetonitrile containing 0.1% *v*/*v* trifluoroacetic acid. Fractions eluting between 10 and 40 min were collected and analyzed by SDS-PAGE to evaluate the purity of the target protein (Figure 2). The identities of the other HPLC peaks were not directly assigned; based on their absence or lower abundance of the expected mouse FZD8 CRD band on SDS-PAGE, they were interpreted as non-target species, likely including misfolded, aggregated, degraded, or impurity-containing fractions. Based on SDS-PAGE densitometry of Figure 2B, the purity of the HPLC-purified FZD8 CRD fraction was estimated to be ~87%. Fractions containing purified FZD8 CRD at 32 min were pooled, dialyzed into 50 mM phosphate buffer at pH 6.5, and concentrated for NMR experiments. From the 6 L culture of ^15^N-labeled FZD8 CRD, approximately 30 mL of refolded and purified sample at ~10 µM in 50 mM phosphate buffer at pH 6.5 was obtained at the final stage of the refolding workflow. The sample was subsequently concentrated as needed, and approximately 40–200 μM FZD8 CRD samples were used to collect the two-dimensional and three-dimensional NMR spectra (SI Figure S3). A two-dimensional ^1^H–^15^N HSQC spectrum was then used as an initial quality-control experiment to assess whether the purified protein adopted a folded, conformationally homogeneous structure [17]. The well-dispersed signals observed in the ^1^H–^15^N HSQC spectrum indicated that the FZD8 CRD was folded (Figure 3 and SI Figure S2). The signals in the ^1^H–^15^N HSQC spectrum of the FZD8 CRD were assigned using a series of NMR experiments (SI Figure S3), as described in Section 4.

## 3. Discussion

This FZD8 CRD refolding workflow can be viewed as a specialized extension of conventional inclusion-body refolding methods. Standard protocols frequently rely on dilution or dialysis to remove denaturants and enable folding, but such approaches may be insufficient for cysteine-rich proteins because productive folding depends on simultaneous control of protein concentration, oxidation state, and disulfide formation [19,20,21]. The present protocol addresses these variables by combining solubilization in high-concentration urea (8 M) and 100 mM β-mercaptoethanol, large-volume dilution into an arginine- and GSSG-containing refolding buffer, concentration at low temperature, dialysis into an acidic solution, lyophilization, and final HPLC purification.

Urea disrupts hydrogen-bonding networks and non-native hydrophobic interactions that stabilize inclusion-body aggregates [19,20,21], thereby promoting unfolding and solubilization of recombinant mouse FZD8 CRD. β-Mercaptoethanol reduces non-native intra- and intermolecular disulfide bonds, effectively resetting the cysteine residues before oxidative refolding. Upon rapid dilution into the refolding buffer, the reduced protein concentration decreases intermolecular collision frequency, whereas arginine suppresses nonspecific protein–protein interactions that can lead to aggregation of partially folded intermediates. GSSG provides an oxidizing environment that promotes disulfide-bond formation during refolding. Subsequent dialysis into acidic solution likely facilitates precipitation of misfolded or aggregated species, allowing their removal before lyophilization and high-resolution HPLC purification.

In this workflow, HPLC purification appeared to serve as more than a final polishing step and functioned as a key enrichment step, providing the resolution needed to enrich a folded, conformationally homogeneous mouse FZD8 CRD species from misfolded and aggregated forms. The supporting NMR spectrum directly illustrates the effect of HPLC purification on the quality of the mouse FZD8 CRD sample (SI Figure S2). Before HPLC, the lyophilized sample dissolved in water showed broad, centrally clustered resonances, consistent with a heterogeneous mixture of folded, misfolded, and/or aggregated species (SI Figure S2A). After HPLC purification, the spectrum became well dispersed, indicating the enrichment of a folded, conformationally homogeneous mouse FZD8 CRD preparation (SI Figure S2B).

During refolding, a native-like folded species is expected to represent one of the most stable conformations and, in principle, should become enriched under favorable refolding conditions. However, because mouse FZD8 CRD contains multiple cysteine residues, many non-native disulfide-bonding patterns can also form during oxidative refolding, and misfolded conformers may become trapped by incorrect disulfide bonds. Therefore, separation after refolding requires a high-resolution purification method capable of distinguishing native-like CRD species from closely related misfolded disulfide-bonded species. Reverse-phase HPLC is more commonly used for peptide and unfolded protein purification than for folded protein purification because organic solvents, such as acetonitrile, can perturb or partially unfold protein structures [33,34]. In this study, however, the FZD8 CRD contains multiple intramolecular disulfide bonds that are not expected to be cleaved by the organic solvent conditions used during HPLC. Thus, although exposure to organic solvents may transiently affect the conformation of the CRD, removal of the solvent by dialysis allows the disulfide-bond-locked protein to recover a folded state, as supported by the well-dispersed NMR spectrum of the HPLC-purified sample (Figure 3 and SI Figures S2 and S3) [34]. However, its application to folded globular proteins is more limited because acidic mobile phases and organic solvents can disrupt noncovalent interactions, alter conformation and oligomeric state, or reduce biological activity. Therefore, when RP-HPLC is used for proteins intended for structural or functional studies, removal of organic solvent, buffer exchange, and independent verification of folding are essential [33,34].

Compared with a simple dilution-only method, additional concentration, dialysis, lyophilization, chromatographic purification, and SDS-PAGE verification steps provide multiple opportunities to remove misfolded or aggregated species and enrich the folded CRD population. Lyophilization was included not only to concentrate the large-volume dialyzed sample and prepare it for HPLC loading but also to provide a practical handling step before final purification. We initially attempted to purify the refolded sample by FPLC before lyophilization; however, FPLC purification alone did not sufficiently resolve the folded CRD species from other species, and RP-HPLC was still required to isolate the folded protein. In addition, lyophilization enabled the partially purified sample to be stored as a dried material at frozen temperatures for extended periods. When additional experiments were needed, the dried sample could be redissolved in water, clarified by centrifugation to remove insoluble aggregated material, and subjected to RP-HPLC analysis or purification. Thus, lyophilization appeared to serve both as a concentration/storage step and as a practical enrichment step, facilitating the removal of residual insoluble aggregates after reconstitution.

Because this study did not systematically omit or vary individual steps, the relative contribution of each step to final recovery and folding could not be quantitatively assigned. The roles of solubilization/reduction, dilution/refolding, acidic dialysis, lyophilization, and RP-HPLC were therefore interpreted from the observed changes in solubility, precipitation behavior, SDS-PAGE fraction analysis, and NMR spectral quality rather than from a formal step-by-step optimization study. Importantly, this refolding workflow was successfully repeated in more than 5 independent preparations, consistently yielding folded FZD8 CRD samples with well-dispersed NMR spectra. These repeated preparations support the reproducibility of the protocol. The complete workflow, from solubilization of inclusion bodies to HPLC purification and preparation of the final folded sample, typically requires approximately one week. Despite its reproducibility, the workflow has practical limitations in scalability and recovery because it requires large-volume dilution, concentration, acidic dialysis, lyophilization, and semi-preparative RP-HPLC. In addition, removing insoluble aggregates and misfolded species during centrifugation, dialysis, and HPLC purification improves the quality of the final folded sample but reduces overall recovery. Overall, the procedure remains complicated and time-consuming, and future optimization may improve its efficiency.

A limitation of this study is that the exact disulfide-bond connectivity of the purified mouse FZD8 CRD was not directly determined by mass spectrometry or peptide mapping. Although mass spectrometry was attempted, it was unsuccessful because the CRD adopts a compact, tightly folded structure stabilized by multiple disulfide bonds, which limited proteolytic digestion and peptide recovery for disulfide-bond analysis. Therefore, the NMR data should be interpreted as evidence for a folded and conformationally homogeneous CRD preparation, rather than definitive proof of the native disulfide-bond connectivity. In addition, we did not perform a direct biological functional assay with the refolded protein. One potential approach to validating the native-like conformation of the refolded FZD8 CRD would be to directly examine its interaction with Wnt proteins. However, with the exception of Wnt3a, most Wnt proteins are highly unstable and cannot be readily purified in sufficient quality or quantity for such binding studies, making this approach technically challenging [35,36]. Nevertheless, purified folded FZD8 CRD has previously been used for structure-guided identification of small-molecule inhibitors of Wnt signaling [17]. The successful use of isolated FZD8 CRD as a platform for discovering Wnt signaling modulators provides indirect support for the notion that the purified CRD can adopt a functionally relevant, native-like conformation [17,18].

**Figure 4:**
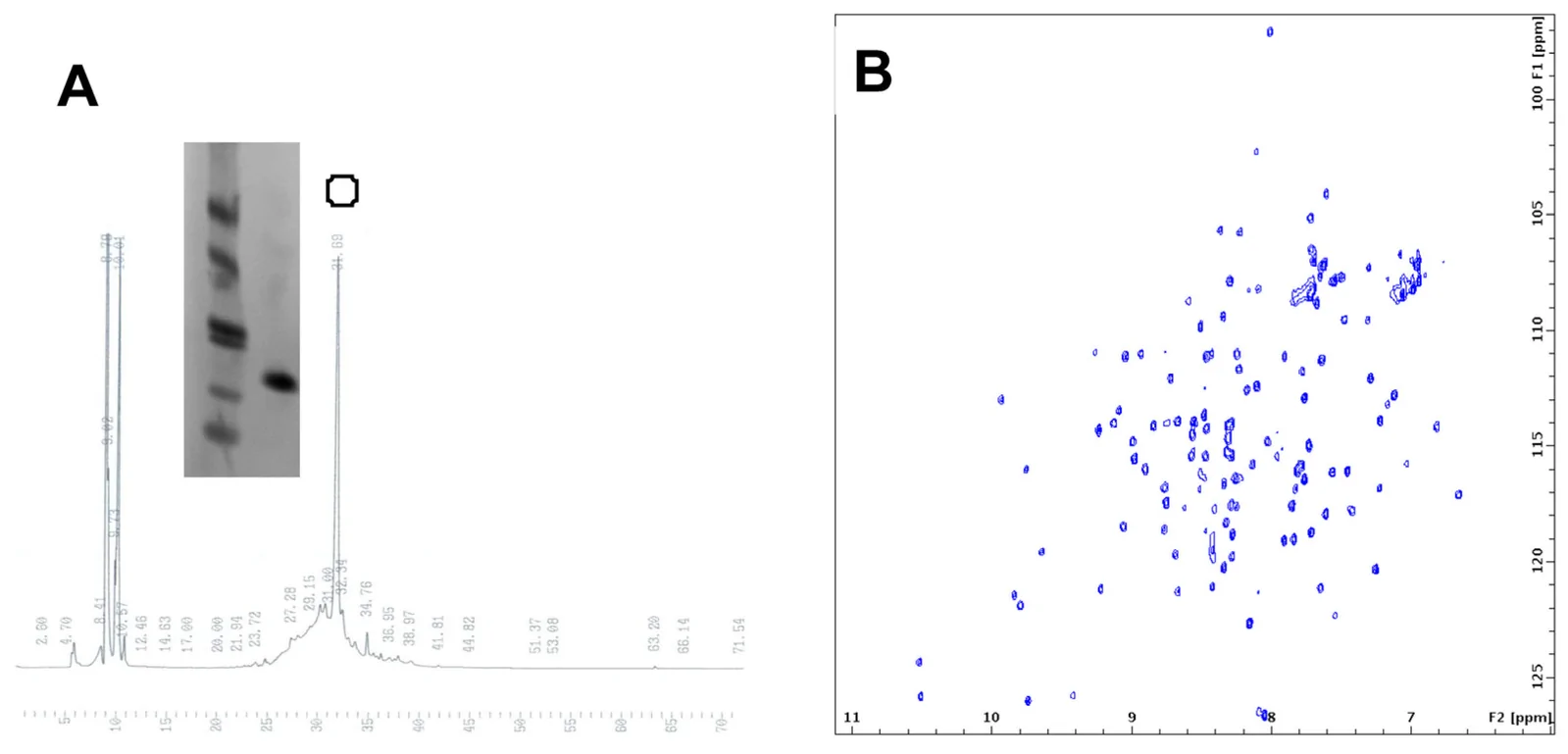
HPLC purification and NMR characterization of mouse sFRP3 CRD (Gly^29^–Ala^152^): (**A**) HPLC chromatogram of the refolded mouse sFRP3 CRD sample after dialysis and lyophilization, showing the elution profile used to identify fractions for analysis. SDS-PAGE analysis of the HPLC fraction collected at approximately 32 min confirms the presence of purified mouse sFRP3 CRD in this fraction. (**B**) Two-dimensional ^1^H–^15^N HSQC spectrum of purified and refolded mouse sFRP3 CRD acquired in 50 mM phosphate buffer at pH 6.5. The well-dispersed HSQC signals indicate that purified mouse sFRP3 CRD adopts a folded conformation. The peak assignments of mouse sFRP3 CRD are shown in Supporting Information Figure S5.

Like mouse FZD8 CRD (Ala^28^–Asp^155^), mouse sFRP3 CRD (Gly^29^–Ala^152^) contains 10 cysteine residues and was expected to be compatible with the refolding strategy developed for mouse FZD8 CRD. Although the two CRDs share only moderate sequence similarity [15], their conserved cysteine pattern and related Frizzled-type CRD architecture suggested that the mouse sFRP3 CRD could serve as an appropriate comparison protein for testing the broader applicability of the protocol. To test this possibility, recombinant mouse sFRP3 CRD expressed in inclusion bodies was subjected to the same refolding workflow established for mouse FZD8 CRD (Figure 4; SI Figures S4 and S5). The resulting mouse sFRP3 CRD preparation was evaluated using the same NMR characterization approach applied to mouse FZD8 CRD. These results indicate that the refolding method developed for mouse FZD8 CRD can also be applied to mouse sFRP3 CRD and support the broader utility of this protocol for cysteine-rich domains.

## 4. Materials and Methods

### 4.1. Protein Expression of Mouse FZD8 CRD and Mouse sFRP3 CRD

The mouse FZD8 CRD construct (Ala^28^–Asp^155^) and the mouse sFRP3 CRD construct (Gly^29^–Ala^152^) were subcloned into the pET28a expression vector and separately transformed into *Escherichia coli* Rosetta2 (DE3) cells (Novagen, London, UK) for recombinant protein expression, respectively (SI Figures S1 and S4) [17]. The expressed FZD8 CRD and sFRP3 CRD constructs contained an N-terminal His-tag followed by a thrombin cleavage site, as indicated by the N-terminal sequence MGSSHHHHHHSSGLVPRGSH. Although the His-tag was present in the FZD8 CRD construct, affinity purification using the His-tag was not performed in this study because the recombinant protein was recovered from inclusion bodies and purified by reverse-phase HPLC after refolding. For isotopically labeled protein production, cells were grown in MOPS minimal medium supplemented with [^15^N]-ammonium chloride and [^13^C]-glucose as the nitrogen and carbon sources, respectively. Uniformly ^13^C/^15^N-double-labeled FZD8 CRD samples were prepared under these conditions for NMR experiments (SI Figure S3). Cultures were grown at 37 °C with shaking at 200 rpm to an A_600_ of 0.5–0.6 and induced with 1 mM isopropyl β-D-1-thiogalactopyranoside. Following induction, cells were incubated for 16 h at 37 °C with shaking at 200 rpm. Under these conditions, the target proteins were predominantly expressed in insoluble inclusion bodies.

### 4.2. Isolation and Solubilization of Inclusion Bodies

After expression of mouse FZD8 CRD (SI Figure S1) or mouse sFRP3 CRD (SI Figure S4), bacterial cells were harvested by centrifugation and resuspended in lysis buffer (20 mM tris, pH 8.0). The cell suspension was disrupted by sonication to lyse the cells and release insoluble recombinant protein aggregates. The lysate was then centrifuged to separate the insoluble material from the soluble fraction (13,000 rpm for 1 h), and the inclusion-body fraction containing mouse FZD8 CRD or mouse sFRP3 CRD was collected as a pellet. The inclusion-body pellet was resuspended in denaturing buffer containing 8 M urea and 100 mM β-mercaptoethanol (Sigma, St. Louis, MO, USA). The suspension was incubated at room temperature with mixing to promote complete denaturation and reduction. Insoluble debris remaining after solubilization was removed by centrifugation, and the clarified supernatant containing denatured and reduced mouse FZD8 CRD or mouse sFRP3 CRD was used for refolding.

### 4.3. Dialysis, Lyophilization, and HPLC Purification

The concentrated refolding mixture (~100 mL) obtained by pressure-driven stirred ultrafiltration (Amicon^®^ Stirred Cells, Merck, Darmstadt, Germany) was dialyzed against 5% acetic acid (Sigma) at 4 °C overnight. Precipitated aggregates and misfolded species after dialysis were removed by centrifugation (4000 rpm for 20 min). The clarified solution was frozen and lyophilized overnight using a lyophilizer, and the lyophilized material was dissolved in 40 mL of water. A portion of the lyophilized material remained insoluble after dissolution in water, likely representing misfolded and/or aggregated species. The suspension was centrifuged, and a 5 mL aliquot of the clarified supernatant was injected onto a ZORBAX 300SB-C18 semi-preparative column (Agilent, Santa Clara, CA, USA; part no. 880995-202; 9.4 × 250 mm, 5 µm particle size, 300 Å pore size; fully porous StableBond C18 stationary phase) for reverse-phase HPLC purification. For reverse-phase HPLC separation of mouse FZD8 CRD or sFRP3 CRD, solvent A was water containing 0.1% *v*/*v* trifluoroacetic acid (TFA), and solvent B was acetonitrile containing 0.1% *v*/*v* TFA. The reverse-phase HPLC run was performed at a flow rate of 2 mL/min, and protein elution was monitored at 260 nm. The HPLC system was programmed with a linear gradient in which the proportion of solvent B was progressively increased, while the proportion of solvent A was correspondingly decreased over the course of the run. During elution, fractions were collected manually at 2 min intervals. The collected fractions were analyzed by SDS-PAGE to identify those containing either the FZD8 CRD or the sFRP3 CRD and to assess the degree of purification (Figure 2 and Figure 4). Fractions containing the target protein were pooled and used for subsequent structural characterization and small-molecule development studies [17].

### 4.4. NMR Characterization and Resonance Assignment of Folded FZD8 CRD

NMR spectra were acquired using Bruker 600 MHz and 800 MHz NMR spectrometers (Bruker BioSpin GmbH, Ettlingen, Germany) located at St. Jude Children’s Research Hospital. For assignment experiments, uniformly ^15^N-labeled or uniformly ^13^C/^15^N-labeled mouse FZD8 CRD was prepared using the same expression, refolding, and purification workflow described above. Initial assessment of sample quality was performed using two-dimensional ^1^H–^15^N HSQC spectra, in which well-dispersed backbone amide resonances were used as an indicator of a folded, conformationally homogeneous CRD. Sequence-specific backbone assignments were obtained using standard three-dimensional HNCA, HNCOCA, CACB(CO)NH, HNCACB, and ^15^N NOESY (mixing time = 120 ms) NMR experiments at 32 °C (Supporting Information Figure S3). NMR data were processed using Bruker Topspin 3.0, and resonance assignments were performed using CARA 1.9 [32] and Sparky 3.115 [32].

## 5. Conclusions

We describe a practical refolding and purification strategy for producing mouse FZD8 CRD from *E. coli* inclusion bodies. Because mouse FZD8 CRD contains ten conserved cysteine residues, recovery of a native-like CRD fold likely requires formation of an appropriate intramolecular disulfide-bonding pattern. The protocol presented here combines denaturant-mediated solubilization, reduction of non-native disulfide bonds, rapid dilution into an arginine-containing glutathione redox buffer, concentration, dialysis, lyophilization, and HPLC purification to enrich folded CRD species while removing aggregated or misfolded forms. Our results also support the notion that, although exposure to organic solvents during reverse-phase HPLC may transiently affect protein conformation, the intramolecular disulfide bonds of a multi-disulfide-bond-containing protein such as CRD are not expected to be disrupted under these conditions. Once the HPLC-purified protein is redissolved in aqueous solution, the disulfide-bond-stabilized CRD can recover a folded state, as supported by the well-dispersed NMR spectrum of the purified protein. This workflow was successfully applied to both mouse FZD8 CRD and mouse sFRP3 CRD, suggesting that it may be useful for other Frizzled-type CRDs that share a conserved cysteine-rich architecture, including those found in smoothened (SMO) and receptor tyrosine kinase-like orphan receptors ROR1 and ROR2 [37,38]. However, further validation will be required before extending this workflow to more distantly related cysteine-rich proteins. Overall, this approach provides a practical route for producing folded CRD samples suitable for NMR spectroscopy, biochemical assays, and ligand-discovery applications [17].

## Data Availability

The original contributions presented in this study are included in the article/Supplementary Materials. Further inquiries can be directed to the corresponding authors.

## Supplementary Materials

The following supporting information can be downloaded at https://www.mdpi.com/article/10.3390/ijms27167198/s1.
