# Supplementary material for: Refolding Protocol of the Cysteine-Rich Domain of Frizzled8 from Inclusion Bodies
Source: Int J Mol Sci. 2026 Aug 12;27(16):7198. doi: 10.3390/ijms27167198 (PMC13513102; doi:10.3390/ijms27167198)
Supplement: Supplementary file 1 [file ijms-27-07198-s001.zip › ijms-4455431-supplementary.pdf]

Supporting Information

Refolding Protocol of the Cysteine-Rich Domain of Frizzled8 from Inclusion Bodies

Ho-Jin Lee [1,*] and Jie J. Zheng [2,3,*]

1. Department of Chemistry, School of Science and Technology, LeMoyne-Owen College, Memphis, TN 38126, USA

2. Jules Stein Eye Institute, Department of Ophthalmology, David Geffen School of Medicine, University of California, Los Angeles, CA, USA

3. Molecular Biology Institute, University of California, Los Angeles, CA, USA

* Correspondence: hjinlab@hotmail.com or ho-jin_lee@loc.edu (H.-J.L.); jzheng@jsei.ucla.edu (J.J.Z.)

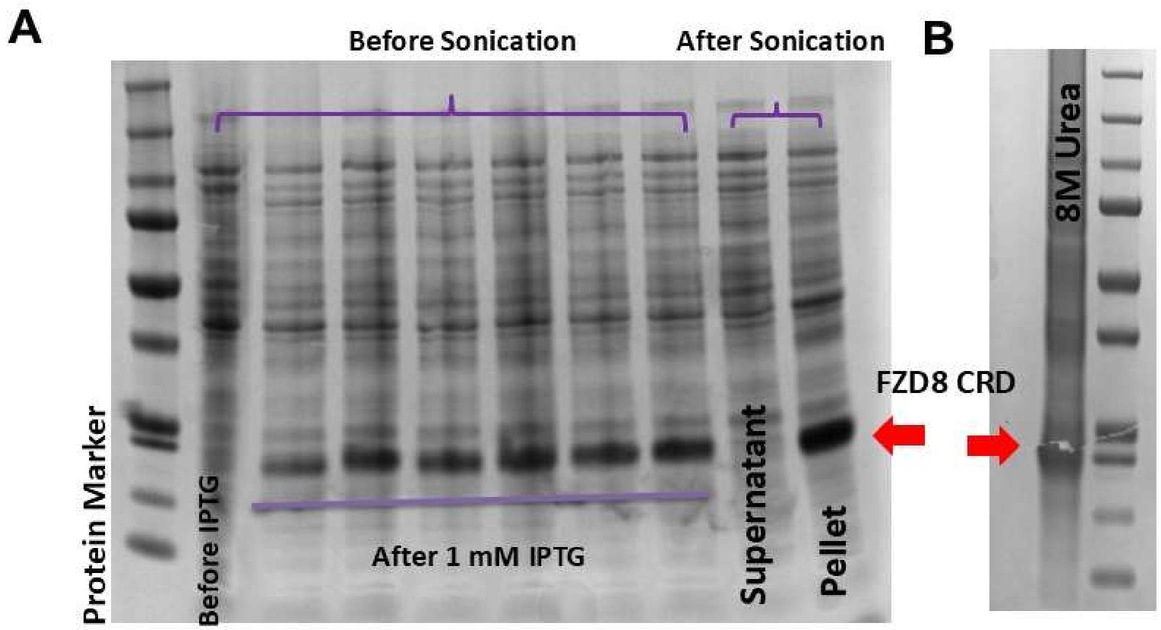

Figure S1. Expression of FZD8 CRD in *E. coli*. SDS-PAGE analysis of ^{15}N-labeled FZD8 CRD expression in *E. coli*. (A) Lane 1, protein marker, in which the bottom band is 10 kDa, followed by the 15 and 20 kDa bands; lane 2, before IPTG induction; lanes 3–8, after induction with 1 mM IPTG from a 6 L culture. The final two lanes show the soluble and insoluble fractions after sonication and centrifugation, indicating that FZD8 CRD is predominantly expressed as inclusion bodies. (B) Soluble fraction obtained after resuspension of the pellet in 8 M urea. The FZD8 CRD construct comprises the sequence MGSSHHHHHH SSGLVPRGSH MASAKELACQ EITVPLCKGI GYNYTYMPNQ FNHDTQDEAG LEVHQFWPLV EIQCSPDLKF FLCSMYTPIC LEDYKKPLPP CRSVCERAKA GCAPLMRQYG FAWPDRMRCD RLPEQGNPDT LCMDYNRTD, with an expected molecular mass of 16.6 kDa.

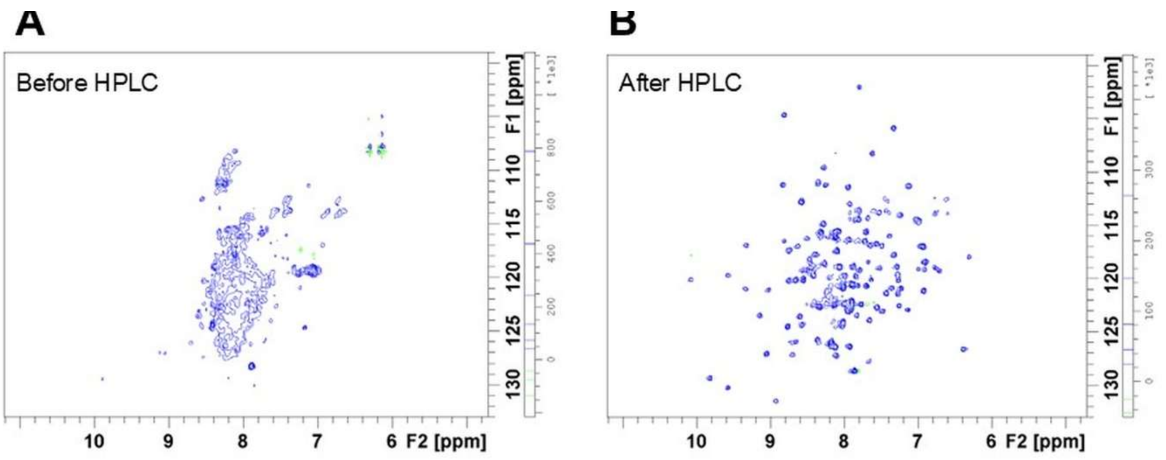

Figure S2. NMR comparison of FZD8 CRD before and after HPLC purification. (A) Before HPLC purification, the NMR spectrum of 15N FZD8 CRD showed broad, poorly resolved signals clustered near the center, suggesting conformational heterogeneity and/or aggregation. (B) After HPLC purification, the spectrum displayed well-dispersed resonances, consistent with enrichment of folded FZD8 CRD. These results demonstrate that HPLC purification is an important step for isolating the properly folded CRD species from the refolded sample.

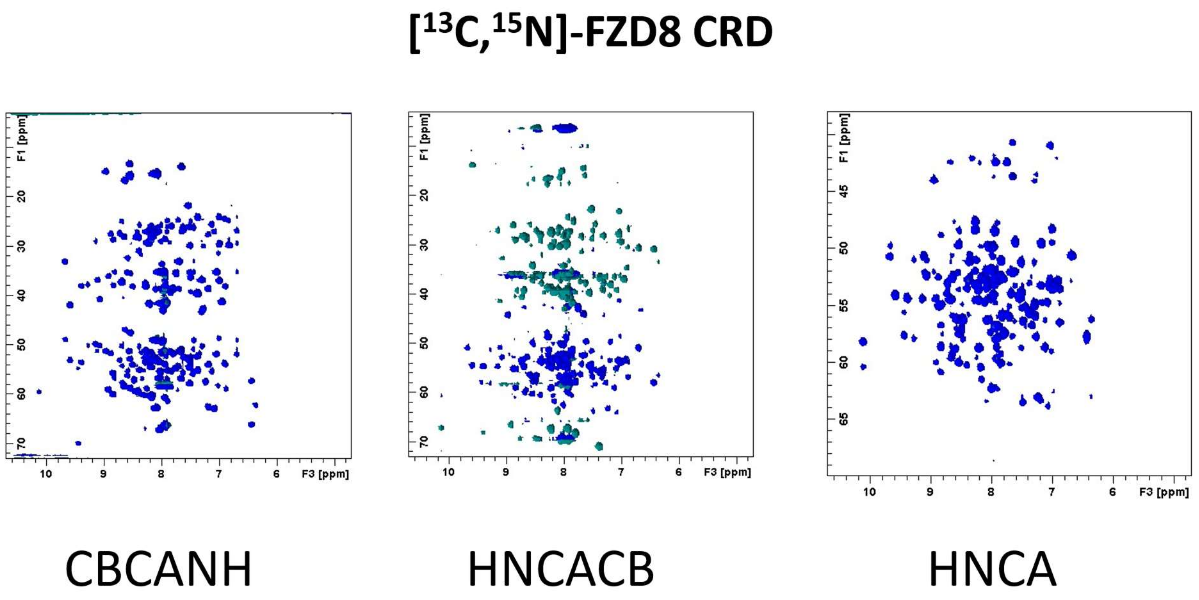

Figure S3. NMR spectra of [^{13}C,^{15}N]-FZD8 CRD. Representative triple-resonance NMR spectra (CBCANH, HNCACB, and HNCA) of a ~200 µM [^{13}C,^{15}N]-FZD8 CRD sample, recorded at 32 °C in 20 mM phosphate buffer, 0.5 mM EDTA, pH 6.5. Spectra were processed and plotted using Bruker Topspin 3.0.

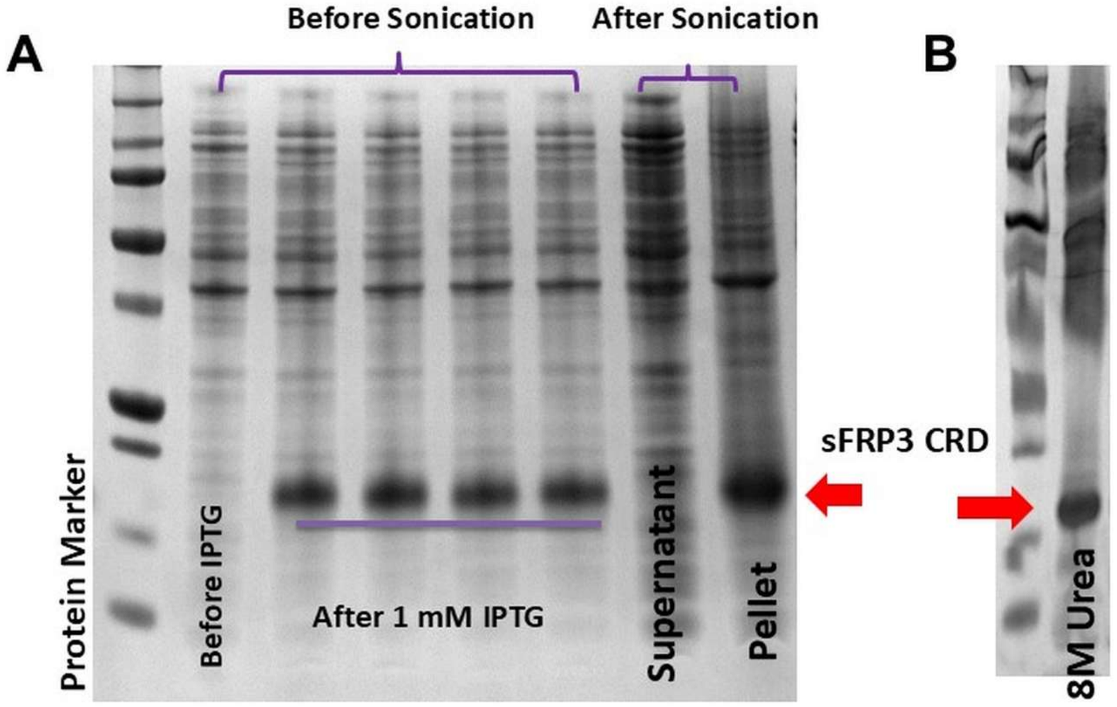

Figure S4. Expression of sFRP3 CRD in *E. coli*. SDS-PAGE analysis of ^{15}N-sFRP3 CRD expression in *E. coli*. (A) Lane 1, protein marker; lane 2, before IPTG induction; lanes 3–6, after induction with 1 mM IPTG, showing that FZD8 CRD is expressed from a 4 L culture. The final two lanes show the supernatant and pellet obtained after sonication and centrifugation, indicating that sFRP3 CRD precipitates as inclusion bodies. (B) Supernatant obtained after solubilizing the pellet in 8 M urea.

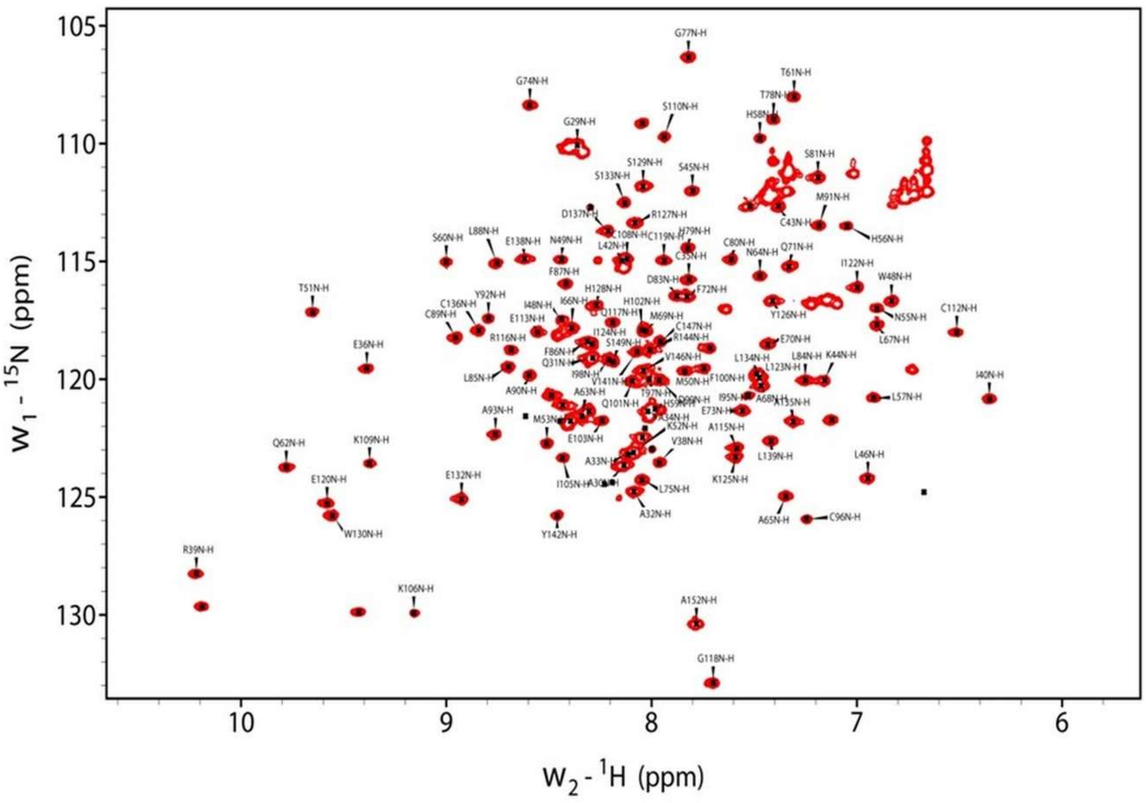

Figure S5. Two-dimensional ^{1}H–^{15}N HSQC spectrum of purified mouse sFRP3 CRD. The spectrum was acquired in 50 mM phosphate buffer at pH 6.5 and shows well-dispersed amide resonances, consistent with a folded sFRP3 CRD structure. NMR data were processed using Bruker Topspin 3.0, and peak assignments were analyzed using CARA (computer-aided resonance assignment) [30] and Sparky [31].
